# Evaluation of High-Dose Isoniazid in Multidrug-Resistant Tuberculosis Treatment

**DOI:** 10.3201/eid3103.241473

**Published:** 2025-03

**Authors:** Valentina Gerussi, Tania Petersen, Isabelle Bonnet, Alexandra Aubry, Marwa Bachir, Esther Gyde, Florence Morel, Corentin Poignon, Brigitte Rached, Valérie Pourcher, Christophe Rioux, Dorothée Vallois, Nicolas Veziris, Jérôme Robert, Lorenzo Guglielmetti

**Affiliations:** Università degli Studi di Trieste, Trieste, Italy (V. Gerussi); Sorbonne Université, Centre National de Référence des Mycobactéries et de la Résistance des Mycobactéries aux Antituberculeux, Assistance Publique–Hôpitaux de Paris, Paris, France (T. Petersen, I. Bonnet, A. Aubry, E. Gyde, F. Morel, C. Poignon, B. Rached, V. Pourcher, N. Veziris, J. Robert, L. Guglielmetti); Centre Hospitalier de Saint-Denis-Centre Hospitalier de Gonesse, Saint-Denis, France (M. Bachir); Université Paris Cité, Paris (C. Rioux); Centre Hospitalier de Bligny, Briis-sous-Forges, France (D. Vallois)

**Keywords:** tuberculosis and other mycobacteria, bacteria, respiratory infections, MDR TB, isoniazid, inhA, katG, mutation, antimicrobial resistance, drug susceptibility testing, phenotypic, genotypic, hepatotoxicity, SCR

## Abstract

High-dose isoniazid is recommended to treat multidrug-resistant tuberculosis (MDR TB). Among 958 MDR TB isolates identified in France during 2008–2022, 93.1% exhibited high-level isoniazid resistance, and molecular testing showed limited diagnostic accuracy in predicting resistance. Clinicians should reconsider using high-dose isoniazid in MDR TB treatment because of suboptimal effect and toxicity concerns.

Despite the turning point represented by the 2022 update in World Health Organization guidelines (*1*), optimal treatment for multidrug-resistant tuberculosis (MDR TB), defined by isoniazid and rifampin resistance, remains a global challenge. The 6-month, all-oral regimen combining bedaquiline, pretomanid, linezolid, and moxifloxacin represents a breakthrough in MDR TB treatment; however, its adoption is limited by cost and access issues. Alternative treatments include the 9-month, all-oral short-course regimen (SCR) and the 18-month, individualized conventional regimen. The SCR is recommended for patients with MDR TB without fluoroquinolone resistance and includes high-dose isoniazid of 10–15 mg/kg. The conventional regimen may also incorporate high-dose isoniazid if there is no confirmation of high-level isoniazid resistance (*1*). The effectiveness of high-dose isoniazid relies on the absence of mutations known to confer phenotypic high-level isoniazid resistance, notably mutations in the *katG* gene (*2*). The high prevalence of such mutations among MDR TB isolates makes the role of high-dose isoniazid in MDR TB regimens questionable (*3*). We quantified the prevalence of high-level isoniazid resistance among MDR TB isolates, particularly isolates from patients eligible for SCR, and evaluated the diagnostic accuracy of molecular testing for predicting high-level isoniazid resistance.

We used data from the comprehensive French national network of the National Reference Centre for Mycobacteria (Paris, France) to perform a retrospective, observational cohort study. We included the isolate obtained at diagnosis from each patient with confirmed pulmonary or extrapulmonary MDR TB identified in France during 2008–2022. We excluded isolates lacking phenotypic drug susceptibility testing (DST) results for isoniazid (0.2 and 1.0 µg/mL) or genotypic data for *inhA*, *katG*, and *gyrA*. For phenotypic DST, solid and liquid cultures were used interchangeably. Testing of solid cultures used the proportion method on Löwenstein-Jensen media, whereas testing of liquid cultures used mycobacteria growth indicator tubes containing Middlebrook 7H9 broth (BD Difco, https://www.bd.com). We defined resistance levels as low-level (0.2 µg/mL) or high-level (1.0 µg/mL) (*4*). Genotypic DST used GenoType MTBDR*plus* and MTBDR*sl* assays (Bruker, https://www.bruker.com) and Sanger or targeted next-generation sequencing (Deeplex Myc-TB; Genoscreen, https://www.genoscreen.fr) when line-probe assay results were missing or uninterpretable. We interpreted results according to the World Health Organization catalog of resistance-associated genetic variants (*5*). We defined eligibility for SCR as the absence of *gyrA* mutations. 

We assessed the diagnostic accuracy of *katG* mutations (with or without mutation in *inhA*) in predicting high-level isoniazid resistance by using phenotypic DST. In the DST calculation, strains without mutations were excluded because they could not be classified as low-level resistance (*inhA* mutation) or high-level resistance (*katG* with or without *inhA* mutation) in phenotypic-genotypic comparisons. 

Descriptive statistics included frequency analyses for categorical variables and median and interquartile range for quantitative variables. We calculated 95% CIs for proportions. We used Stata 15.2 (StataCorp LLC, https://www.stata.com) for analyses and considered p<0.05 statistically significant. Ethical approval was granted by the ethics review board of the Bligny Hospital, Briis-sous-Forges, France (study design approved by Conseil de Réflexion Ethique on June 27, 2023).

Among 1,089 MDR TB isolates, 958 were included in the study (Figure). Of those isolates, 892 (93.1%, 95% CI 91.5–94.7) exhibited high-level isoniazid resistance (Table). Mutations in *katG* were found in 837 (87.4%) isolates and *inhA* mutations in 259 (27.0%) isolates. Of note, 828 (98.9%) of the 837 isolates with *katG* mutations showed high-level isoniazid resistance. In addition, we detected high-level isoniazid resistance in 51.0% of isolates with *inhA* mutations and without *katG* mutations. Of the 739 isolates from patients eligible for the SCR (Figure), 677 (91.6%) had high-level isoniazid resistance. The diagnostic accuracy of genotypic testing for predicting high-level isoniazid resistance compared with phenotypic DST was as follows: sensitivity 93.3% (95% CI 91.6–94.9), specificity 86.4% (95% CI 84.2–88.6), positive predictive value 99.0% (95% CI 98.4–99.7), and negative predictive value 46.4% (95% CI 43.2–49.6). Those accuracy metrics were comparable among isolates from patients eligible for SCR (Table).

**Figure:**
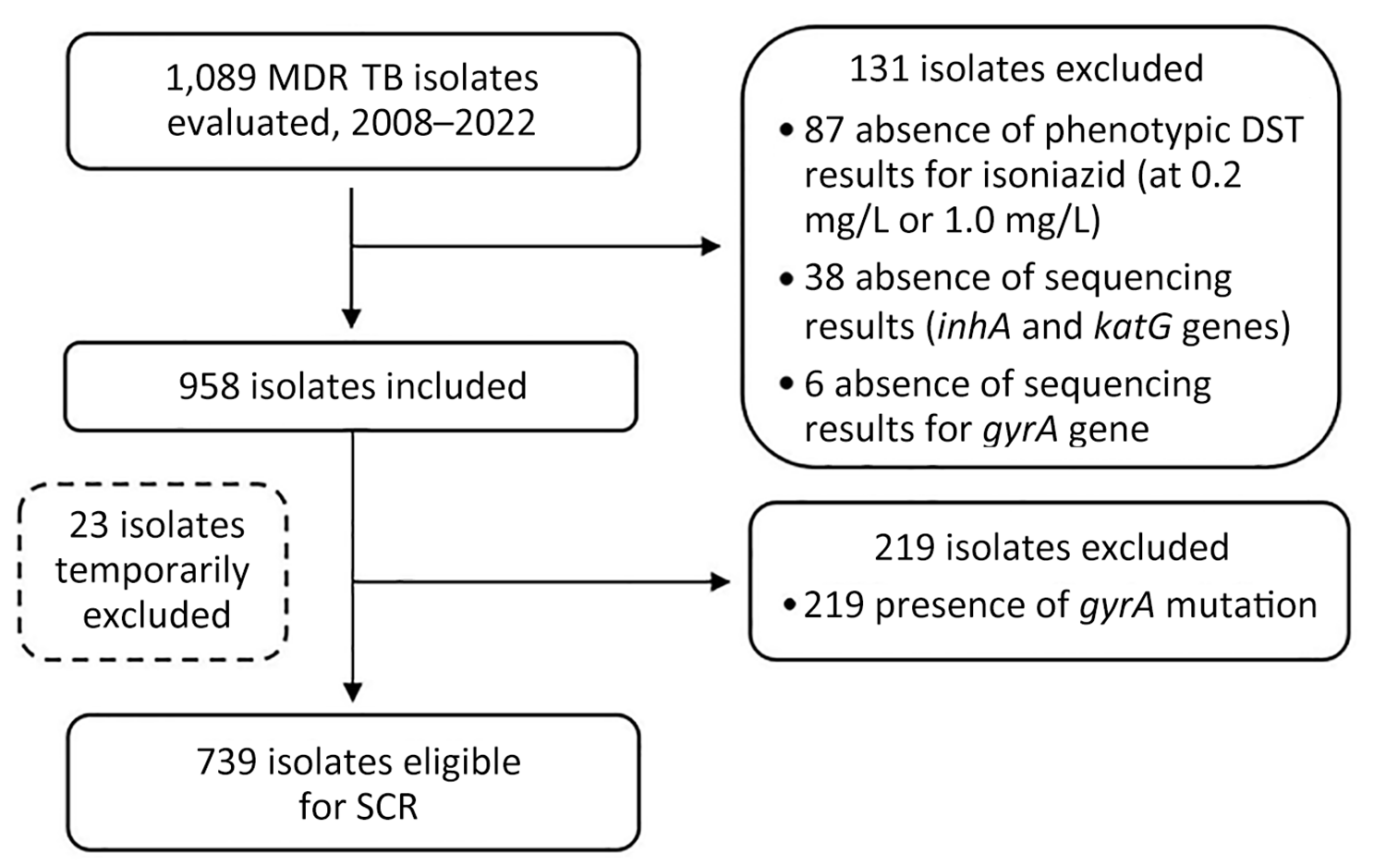
Flowchart of MDR TB isolates identified in France during 2008–2022 included in an evaluation of high-dose isoniazid in MDR TB treatment. DST, drug susceptibility testing; MDR TB, multidrug-resistant tuberculosis; SCR, short-course regimen.

**Table:** Phenotypic and genotypic methods for MDR TB isolates identified in France during 2008–2022 used in evaluation of high-dose isoniazid use in MDR TB treatment*

Isoniazid resistance	All isolates, n = 958	Isolates from patients eligible for SCR, n = 739
Phenotypic isoniazid resistance		
High-level resistance†	892 (93.1; 91.5–94.7)	677 (91.6; 89.6–93.6)
Low-level resistance‡	66 (6.9; 5.3–8.5)	62 (8.4; 6.4–10.4)
Genotypic isoniazid resistance		
*katG* + *inhA* or its promoter mutation	161 (16.8; 14.4–19.2)	104 (14.1; 11.6–16.6)
*katG* mutation alone	676 (70.6; 67.7–73.5)	525 (71.0; 67.8–74.3)
*inhA* or its promoter mutation alone	98 (10.2; 8.3–12.1)	89 (12.1; 9.7–14.4)
No mutation	23 (2.4; 1.4–3.4)	21 (2.8; 1.6–4.0)
Diagnostic accuracy of genotypic testing (*katG* mutation) to predict high-level isoniazid resistance, % (95% CI)		
Sensitivity	93.3 (91.6–94.9)	92.3 (90.4–94.3)
Specificity	86.4 (84.2–88.6)	87.3 (84.8–89.7)
Positive predictive value	99.0 (98.4–99.7)	98.9 (98.1–99.7)
Negative predictive value	46.4 (43.2–49.6)	48.5 (44.8–52.1)

*Values are no. (%; 95% CI) except as indicated. MDR TB, multidrug-resistant TB; SCR, short course regimen. †Defined as resistance to the 1.0 µg/mL dose. ‡Defined as resistance to the 0.2 µg/mL dose.

Our findings indicated that high-dose isoniazid is unlikely to be effective for most patients using the MDR TB regimen because of the high prevalence of high-level isoniazid resistance, including those from patients eligible for SCR. The high frequency of observed *katG* mutations aligns with previous studies; most *katG* mutant isolates exhibited high-level isoniazid resistance (*3*). Furthermore, the absence of *katG* mutations alone does not reliably exclude high-level isoniazid resistance because more than half of strains in our study with *inhA* mutations displayed isoniazid MICs >1.0 mg/L. Although high-dose isoniazid was previously considered effective against *inhA* mutant isolates (*6*), more recent research reported MICs >1.0 mg/L in those strains (*7*,*8*), which limits the utility of genotypic testing in predicting low-level isoniazid resistance. Although high-dose isoniazid may still be appropriate in specific cases, the associated toxicity risks suggest that its inclusion in MDR TB regimens may not be warranted (*9*,*10*).

In summary, high-dose isoniazid offers limited benefit for most patients using the MDR TB regimen because of widespread high-level isoniazid resistance. Clinicians should optimize existing regimens, replace high-dose isoniazid with safer, more effective alternatives, and promote global access to new treatments.
